# Cardiac Chemodectoma With Recurrent Pericardial Effusion and Cardiac Tamponade in an Alaskan Malamute: A Case Report

**DOI:** 10.1155/crve/8575861

**Published:** 2026-10-04

**Authors:** Sachin Patil, Aniket Mhase, Sarvesh Rai, Mukundraj Raut, Akarsh Sharma

**Affiliations:** ^1^ Greens Zoological Rescue and Rehabilitation Centre Jamnagar Gujarat India; ^2^ Khodiyar Animal Welfare Trust Jamnagar Gujarat India

**Keywords:** aortic body tumor, chemodectoma, echocardiography, heart-base tumor, pericardial effusion

## Abstract

Chemodectomas (aortic body tumors) are rare, slow‐growing neoplasms arising from chemoreceptor tissue at the heart base in dogs, most frequently reported in brachycephalic breeds; to the authors′ knowledge, their occurrence in nonbrachycephalic large breeds, such as the Alaskan Malamute, is uncommonly documented. An 8‐year‐old intact male Alaskan Malamute (body weight 44.8 kg) was referred with a history of lethargy, inappetence, exercise intolerance, and ascites. Echocardiography confirmed extensive pericardial effusion with cardiac tamponade and a multinodular, irregularly shaped heart‐base mass invading the left atrium, right atrium, left auricle, and aortic root. The dog underwent two pericardiocentesis procedures, yielding 350 and 630 mL of serosanguinous fluid on Days 1 and 15, respectively. The dog died prior to the scheduled 1‐week re‐evaluation. Postmortem examination confirmed a multinodular, irregularly shaped, extensively invasive mass involving all cardiac chambers and the aortic root, with histopathological features morphologically consistent with chemodectoma. This case documents a fatal cardiac chemodectoma in an Alaskan Malamute, contributing to the limited literature on heart‐base tumors in nonbrachycephalic large breeds, and emphasizes the importance of thorough echocardiographic evaluation, serial imaging in dogs with recurrent pericardial effusion, and counseling regarding a guarded‐to‐poor prognosis with extensive cardiac invasion.

## 1. Introduction

Chemodectoma, also referred to as aortic body tumor or heart‐base tumor, is a neoplasm originating from the chemoreceptor (glomus) cells of the aortic and carotid bodies [1, 2]. In dogs, aortic body tumors are the most common type, arising at the base of the heart near the aortic arch, and are characteristically slow‐growing but locally invasive [3]. Aortic body tumors are predominantly reported in brachycephalic breeds—particularly the Boxer, Boston Terrier, and English Bulldog—and collectively account for approximately 7% of heart tumors in dogs [3]. Chronic hypoxia in brachycephalic dogs is postulated to drive chemoreceptor cell hyperplasia and subsequent neoplastic transformation [2].

Large nonbrachycephalic breeds such as the Alaskan Malamute are considered to be at considerably lower risk than brachycephalic breeds, and to the authors′ knowledge, published reports of cardiac chemodectoma in Alaskan Malamutes are rare. Pericardial effusion is a potentially life‐threatening complication noted due to aortic body tumor [4, 5]. Echocardiography is the principal noninvasive diagnostic modality for identifying pericardial effusion and characterizing cardiac masses, whereas histopathology and immunohistochemistry remain the gold standard for definitive diagnosis [1, 2, 4].

Beyond the occurrence of this tumor in a nonbrachycephalic breed, the specific novelty of the present case lies in the combination of extensive local invasion involving all four cardiac chambers and the aortic root, a multinodular, irregularly shaped tumor morphology, recurrent hemorrhagic pericardial effusion with cardiac tamponade, and a rapidly fatal clinical course. This particular constellation of features, rather than any single element in isolation, distinguishes the present case and substantially expands the clinicopathological spectrum recognized for heart‐base tumors in veterinary cardiology.

## 2. Case Presentation

### 2.1. Signalment and History

An 8‐year‐old intact male Alaskan Malamute weighing 44.8 kg was referred to the Department of Cardiology, Kingdom of Dogs and Cats, Khodiyar Animal Welfare Trust, Jamnagar, India. The presenting complaint included lethargy, inappetence, exercise intolerance, and abdominal distension. Heart rate at presentation was 120 beats per minute. The dog had no relevant previous medical history and no prior episodes of pericardial effusion. Clinical signs were of acute onset; given the typically slow, insidious progression of heart‐base tumors, it is likely that milder, earlier signs had gone unnoticed before this acute decompensation.

### 2.2. Clinical Examination

Physical examination revealed pale pink mucous membranes with a prolonged capillary refill time (> 3 s); rectal temperature was within normal limits. Thoracic auscultation revealed muffled heart sounds and increased respiratory effort, with a respiratory rate of 45 breaths per minute. Peripheral pulse quality was reduced, and the dog remained exercise intolerant. Abdominal palpation demonstrated distension with a positive fluid thrill, consistent with ascites, which was subsequently confirmed by abdominal ultrasonography (Figure 1).

**Figure 1:**
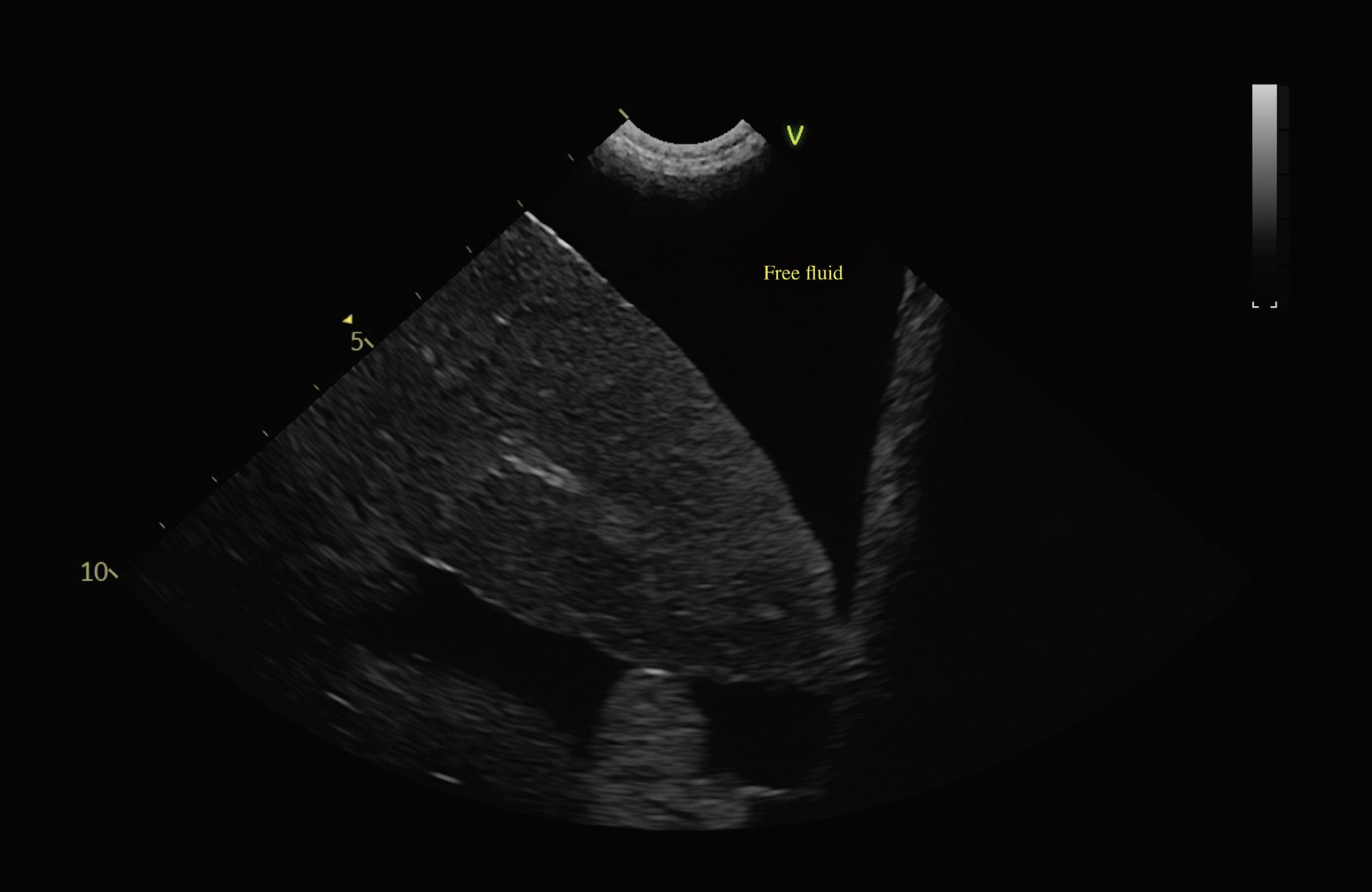
Abdominal ultrasonography at first presentation confirming ascites.

Hematology demonstrated anemia, whereas serum biochemistry revealed hypoalbuminemia (Table 1).

**Table 1:** Hematology and biochemistry at presentation.

Parameter	Result	Reference interval
White blood cells (10^3^/mm^3^)	7.1	6.0–17.0
Lymphocyte (%)	16.3	12–30
Monocyte (%)	4.4	3–10
Neutrophil (%)	79.3	60–77
Eosinophil (%)	2	2–10
Red blood cells (10^6^/mm^3^)	4.42	5.50–8.50
Hemoglobin (g/dL)	8.3	12.0–18.0
Packed cell volume (%)	25.8	37.0–55.0
Platelets (10^3^/mm^3^)	408	200–500
AST (U/L)	27	17–44
ALT (U/L)	23	17–78
ALP (U/L)	32	≤ 89
TP (g/dL)	5.3	5.0–7.2
Albumin (g/dL)	1.9	2.6–4.0
TBIL (mg/dL)	0.2	≤ 0.5
BUN (mg/dL)	14.8	9.2–29.2
Creatinine (mg/dL)	0.96	0.40–1.40

*Note:* Hematological reference intervals were obtained from the manufacturer′s canine reference range documentation for the HORIBA veterinary hematology analyzer, itself based on Schalm′s Veterinary Hematology, 6th edition [6]. Biochemical reference intervals were obtained from FUJIFILM VET Systems Co. Ltd., canine biochemistry reference intervals for the FUJI DRI‐CHEM analyzer platform [7].

### 2.3. First Evaluation: Echocardiography and Pericardiocentesis

Transthoracic two‐dimensional (2D) echocardiography was performed on a GE Vivid iQ ultrasound system (GE Healthcare, Chicago, Illinois, United States) using a phased‐array transducer of frequency 2–5 MHz. Standard imaging planes were obtained including right parasternal long‐axis four‐ and five‐chamber views, right parasternal short‐axis views at the aortic valve and papillary muscle levels, and left apical four‐chamber views. Color‐flow Doppler was used to characterize valvular regurgitation.

Echocardiography demonstrated volume overload of the left atrium and right atrium, which was assessed subjectively (Figure 2A) with hyperkinesia of both ventricles, interventricular septal flattening consistent with right ventricular volume overload, mild mitral regurgitation, severe tricuspid regurgitation, and extensive pericardial effusion. A multinodular, irregularly shaped, extensively invasive heart‐base mass was identified, invading the left atrium, right atrium, left auricle, and aorta (Figure 2A–C). Key echocardiographic measurements are summarized in Table 2.

**Figure 2:**
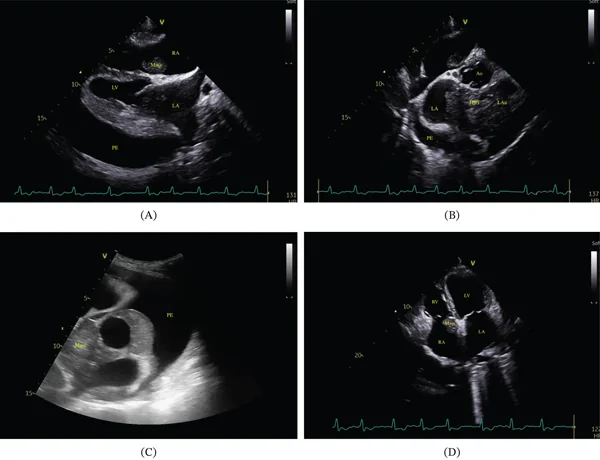
(A) Right parasternal long‐axis four‐chamber echocardiographic view demonstrating marked volume overload of the left atrium (LA) and right atrium (RA), mass in the right atrium, and extensive pericardial effusion (PE). (B) Right parasternal short‐axis echocardiographic view at the level of the aortic root demonstrating heart base tumor (HBT) infiltrating the LA, left auricle (LAu), RA, and surrounding the aorta (Ao). PE is visible in the near field. (C) Ultrasonography view showing tumor mass at the heart base and PE with cardiac tamponade. (D) Echocardiographic image obtained immediately after pericardiocentesis showing reduction in pericardial effusion and improvement in cardiac chamber dimensions.

**Table 2:** Serial echocardiographic measurements with reference values.

Parameter	Exam 1	Exam 2	Reference range
LVIDd (cm)	4.55	3.99	4–5.6
LVIDdN	1.34	1.24	1.63 (upper normal)
LVIDs (cm)	2.84	2.13	2.6–4.1
LVIDsN	0.76	0.61	1.09 (upper normal)
IVSd (cm)	1.12	0.84	0.8–1.5
IVSs (cm)	1.36	1.18	1.1–2
LVWd (cm)	1.42	2.28	0.8–1.4
LVWs (cm)	2.07	2.73	1.2–2
LAD (cm)	5.5	5.24	—
LADN	1.6	1.62	< 1.57
EDV (mL)	124.66	85.56	—
EDVN (mL/kg)	3.47	2.83	1.25–3.27
ESV (mL)	74.94	30.54	—
ESVN (mL/kg)	1.93	0.94	0.3–1.54
LA/Ao	—	1.7	< 1.6

*Note:* Source: [8–12].

Pericardiocentesis was performed with the dog in sternal recumbency. The area from the third to the seventh rib was clipped and aseptically prepared. The site of entry was at the fifth intercostal space, lateral to the sternum and near the costochondral junction. Lignocaine (2%) was infiltrated subcutaneously at the insertion site. A 14‐gauge, 45‐mm intravenous catheter was advanced under continuous echocardiographic guidance. A total of 350‐mL of serosanguinous fluid was drained from the pericardial sac and was sent for laboratory analysis [13]. Clinically evident hemodynamic improvement was observed following drainage (Figure 2D).

Physical examination of pericardial fluid showed hazy appearance, pH 6.5 and specific gravity 1.020; whereas microscopic examination showed RBC count 250,000/mm^3^ and WBC count 1050/mm^3^. Cytological analysis revealed high cellularity with a clear background. The smear contained erythrocytes and leukocytes in normal proportions; among leukocytes, 80% were polymorphonuclear cells and 20% mononuclear cells. No atypical or neoplastic cells and no bacteria were identified.

### 2.4. Second Evaluation: Echocardiography and Pericardiocentesis

The dog was re‐evaluated 15 days after the initial presentation. Body weight had declined to 37.3 kg, representing a loss of 7.5 kg (16.7%) over 15 days. This weight change likely reflects a combination of factors including reduced appetite, fluid losses associated with pericardiocentesis, and possible muscle wasting [14].

Echocardiography using parasternal long‐ and short‐axis views again demonstrated the multinodular, irregularly shaped, extensively invasive heart‐base mass infiltrating the left atrium, right atrium, left auricle, and aortic root. Extensive pericardial effusion had recurred. Volume overload of the right ventricle, right atrium, and left atrium, interventricular septal flattening, severe tricuspid regurgitation, and mild mitral regurgitation persisted. Volume overload in the right heart was assessed subjectively.

Emergency pericardiocentesis was repeated using the technique described above. This procedure yielded 630 mL of serosanguinous fluid, indicating rapid hemorrhagic reaccumulation within 15 days suggestive of recurrent hemorrhagic effusion associated with the heart‐base mass [4, 5].

### 2.5. Clinical Outcome

The dog died prior to the scheduled 1‐week re‐evaluation following the second pericardiocentesis. The rapid recurrence of massive pericardial effusion, progressive cardiac invasion by the tumor, and severe right‐sided heart failure collectively reflected advanced, terminal disease.

### 2.6. Necropsy and Histopathology

A complete postmortem examination was performed. Visceral and pathoanatomical lesions were recorded and representative tissue samples from all major organs were collected in 10% neutral buffered formalin for histopathological examination [15, 16]. Fixed tissues were processed by standard paraffin‐wax embedding, and sections stained with hematoxylin and eosin (H&E) were examined by light microscopy (Nikon Eclipse Si, Nikon Corporation, Tokyo, Japan) and digitally photomicrographed.

Gross findings: The carcass was weak and emaciated with pale mucous membranes. Cardiac tamponade was present, with accumulation of a large volume of reddish fluid in the pericardial sac. A large, firm, multinodular, irregularly shaped neoplastic mass was present at the base of the heart, bulging into both auricles, the ventricles, and the aorta. The left ventricle and aortic root were surrounded by the neoplastic growth. The mass compressed the pulmonary arteries without invading their lumina. On cross‐section, the mass showed a highly vascularized periphery with a mottled red‐to‐brown mosaic pattern (Figure 3). Mild endocardiosis of the mitral and tricuspid valves was noted. The thoracic cavity contained a small amount of reddish fluid. The lungs were mildly congested and edematous. The liver was slightly enlarged with a prominent reticular pattern. No other significant postmortem findings were recorded.

**Figure 3:**
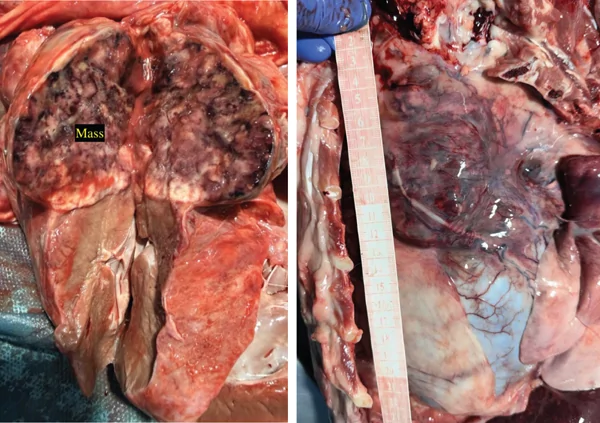
Gross pathology: The heart‐base neoplastic mass showing a mottled red‐to‐brown mosaic pattern with a highly vascularized periphery. The mass is multinodular and irregular in shape.

Histological findings: Histological sections of the cardiac mass revealed clusters of round to oval pleomorphic cells arranged in a disorganized manner in variable‐sized lobules separated by delicate connective tissue strands (Figure 4). Cells exhibited markedly pleomorphic nuclei with distinct nucleoli, moderately eosinophilic granular cytoplasm, and indistinct cell borders. Nuclei were round, oval, or irregular with marked anisokaryosis and karyomegaly. Mitotic figures numbered 1–3 per high‐power field, and binucleated cells were observed. Scattered giant cells with large, bizarre, hyperchromatic nuclei, and degenerated neoplastic cells with pyknotic nuclei were present throughout. Neovascularization was prominent, with multifocal hemorrhage and multifocal to coalescing extensive necrosis centrally. The neoplastic cell nests were extensively invasive, replacing myocardial fibers. The diagnosis of cardiac chemodectoma (aortic body tumor) was made based on gross findings, examination, and histomorphological features. The immunohistochemistry could not be performed in the present study due to laboratory constraints, which could have aided in definitive lineage confirmation.

**Figure 4:**
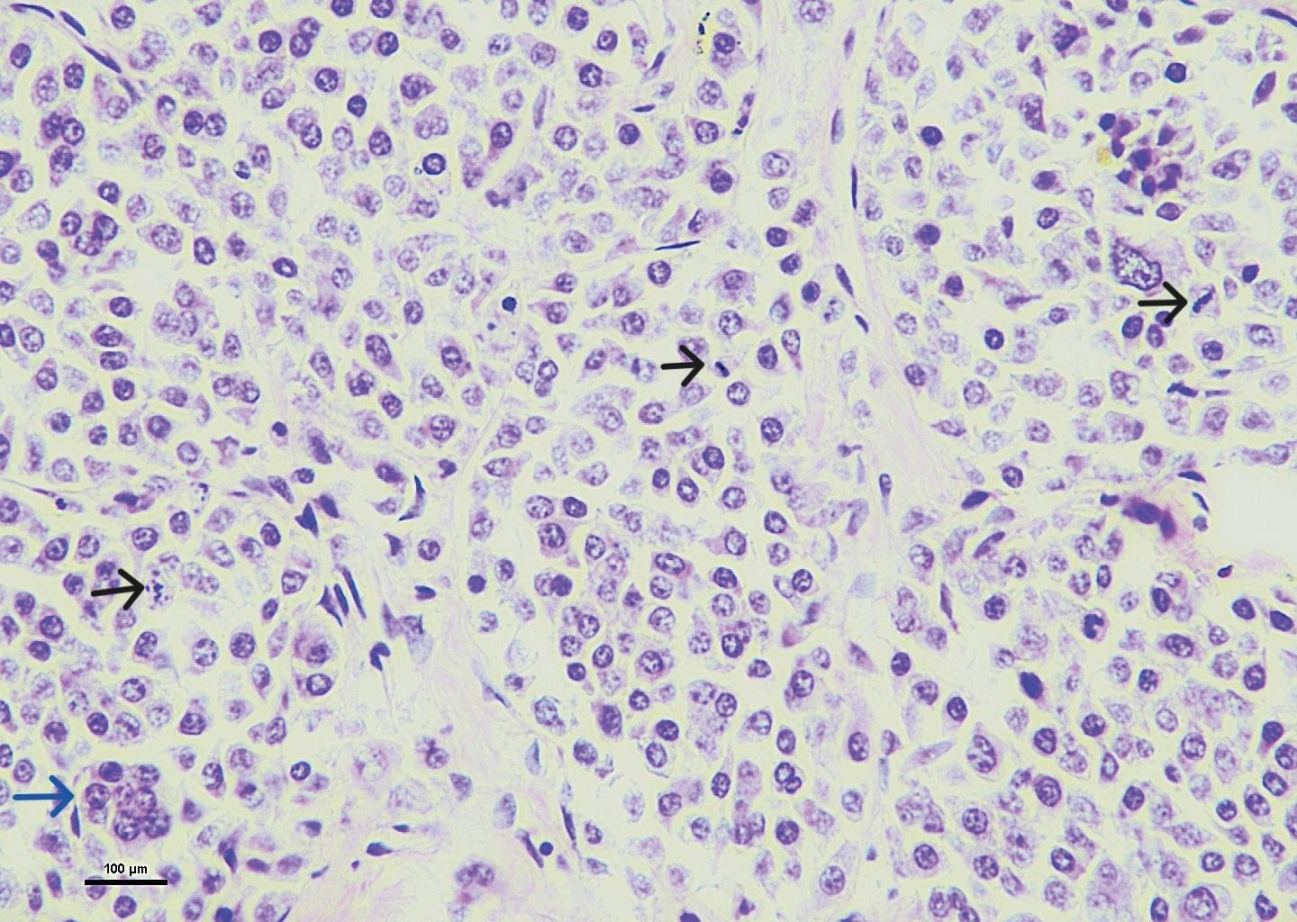
Photomicrograph (H&E, objective lens 40X). Pleomorphic neoplastic cells are present in a disorganized pattern along with multinucleated giant cells (black arrow), binucleated cells, and mitotic figures (blue arrow).

Hepatic sections showed extensive periportal and bridging fibrosis, centrilobular hepatocyte atrophy, degeneration, and necrosis with mononuclear cell infiltration, consistent with congestive hepatopathy [17, 18]. Pulmonary sections revealed severe vascular congestion with focal hemorrhage, interstitial and alveolar edema, and foamy macrophages. Renal sections showed marked tubular degeneration. These findings were interpreted as sequelae of chronic right‐sided heart failure and cardiac tamponade rather than primary organ disease. The representative sections of lung, liver, kidney, spleen, stomach, intestine, brain, and lymph nodes showed no evidence of neoplastic metastasis.

## 3. Discussion

This case documents a fatal cardiac chemodectoma in an Alaskan Malamute—a nonbrachycephalic large breed in which, to the authors′ knowledge, this tumor is uncommonly reported. The clinical course was characterized by rapidly progressive, recurrent hemorrhagic pericardial effusion culminating in cardiac tamponade, progressive right‐sided heart failure, and death within approximately 3 weeks of initial presentation.

Chemodectomas in dogs are most frequently reported in brachycephalic breeds, with the Boxer accounting for the majority of published cases [2, 3, 19]. The proposed pathogenesis involves chronic arterial hypoxia stimulating persistent chemoreceptor cell hyperplasia, with eventual neoplastic transformation [2]. To the authors′ knowledge, nonbrachycephalic large breeds remain at considerably lower risk compared with brachycephalic breeds, and the occurrence of chemodectoma in such breeds is uncommon. The present case adds to a small body of evidence that heart‐base tumors should not be excluded from the differential diagnosis in large nonbrachycephalic breeds presenting with hemorrhagic pericardial effusion [3, 4].

Management of pericardial effusion secondary to cardiac neoplasia is inherently palliative [5, 20]. Pericardiocentesis provides temporary hemodynamic relief but does not address the underlying tumor [5]. Accumulation of pericardial effusion after 15 days of first pericardiocentesis illustrates the aggressive hemorrhagic potential of vascular heart‐base tumors and the rapidity with which life‐threatening re‐accumulation can occur [4, 5].

Surgical pericardiectomy has been advocated as a palliative measure to prolong survival; in a retrospective study of 24 dogs with histologically confirmed aortic body tumors, dogs that underwent pericardiectomy had a reported median survival of approximately 730 days compared with 42 days in those that did not, although treatment was not randomized and dogs selected for pericardiectomy may have represented a more favorable subset with less extensive disease [20]. In the present case, the extensive invasion of the mass into all four cardiac chambers and the aortic root would likely have substantially limited both the feasibility and the potential benefit of pericardiectomy.

Toceranib phosphate (Palladia), a tyrosine kinase inhibitor with reported antiangiogenic and antitumor activity across a range of canine solid tumors, may be considered as an adjunctive medical therapy in cases where surgery is not feasible [21]. Evidence specific to chemodectoma is more limited but growing: A multi‐institutional retrospective study of 27 dogs with presumed or confirmed aortic body chemodectomas treated with toceranib reported an 89% clinical benefit rate and a median toceranib‐specific survival of 478 days [22], consistent with an earlier single case report describing sustained clinical and radiographic improvement in a dog with a confirmed chemodectoma [23]. Nonetheless, this chemodectoma‐specific evidence remains confined to retrospective and single‐case data, and toceranib was not used in the present case.

The tumors most commonly associated with the heart base include hemangiosarcoma, lymphoma, ectopic thyroid carcinoma, and chemodectoma [24]. Hemangiosarcoma is a highly malignant tumor arising from vascular endothelial cells and is the most common primary cardiac tumor of dogs [25]. Histological features of hemangiosarcoma typically include spindle‐shaped cells organized to form cavernous vascular structures [26]. Lymphoma affecting the heart in dogs is uncommon but can cause diffuse or nodular lesions [27]. Follicular ectopic thyroid carcinomas can be readily differentiated histologically by the presence of follicular structures filled with eosinophilic colloid [28]. The histologic differentiation of solid or anaplastic ectopic thyroid carcinomas from chemodectomas is difficult owing to the lack of distinct follicles. However, thyroid specific immunohistochemical marker TTF‐1 (thyroid transcription factor‐1) and thyroglobulin can assist in differential diagnosis of chemodectoma with thyroid carcinoma [28].

Immunohistochemistry is considered the standard for definitive diagnosis of chemodectoma. In the present case, immunohistochemistry was not performed owing to laboratory constraints, and the diagnosis is therefore based on anatomical location of tumor, gross findings, and histomorphological features.

The cytological analysis of pericardial effusion yields definitive diagnosis for identifying underlying lymphoma or infectious etiology. However, neoplastic diagnosis other than lymphoma (chemodectoma, mesothelioma, and hemangiosarcoma) is rarely diagnosed based on cytology. Chemodectomas (heart‐base tumors) are poorly exfoliating; therefore, cytologic evaluation of pericardial effusion does not usually provide conclusive findings. The cytological findings of pericardial effusion in this case are consistent with previous studies [4]. This finding underscores the importance of echocardiographic and, ultimately, histopathological assessment and immunohistochemistry for definitive diagnosis.

The secondary organ pathology, including congestive hepatopathy, was consistent with chronic right‐sided heart failure and confirms the systemic impact of prolonged cardiac tamponade and venous hypertension [17, 18]. The absence of visceral metastasis, despite histological evidence of malignant features including mitotic activity and vascular invasion, is consistent with published observations that chemodectomas are usually locally malignant but can rarely metastasize [1].

To authors′ knowledge, this is one of the few published cases of cardiac chemodectoma in the Alaskan Malamute breed. The case demonstrates that chemodectoma must be included in the differential diagnosis for any large‐breed dog presenting with hemorrhagic pericardial effusion, including in nonbrachycephalic breeds [3, 4]. Clinicians should maintain a high index of suspicion, employ serial and multimodality imaging, and counsel owners regarding the guarded‐to‐poor prognosis associated with extensive cardiac invasion [4, 20].

## 4. Conclusions

This case highlights the diagnostic and therapeutic challenges associated with cardiac chemodectoma in dogs, particularly when complicated by recurrent pericardial effusion and cardiac tamponade. Comprehensive imaging and timely pericardiocentesis provided temporary hemodynamic stabilization and helped characterize the extent of disease, but did not constitute definitive treatment of the underlying neoplastic process. Despite temporary improvement, the recurrence of effusion and the infiltrative nature of the mass underscored the guarded‐to‐poor long‐term prognosis associated with heart‐base tumors. This report reinforces the importance of early recognition, repeated monitoring, and multidisciplinary decision‐making in managing neoplastic pericardial disease in canine patients.

**Nomenclature**
%: percent
×: magnification (microscopy)
2D: two‐dimensional
ALP: alkaline phosphatase
ALT: alanine aminotransferase
Ao: aorta
AST: aspartate aminotransferase
BW: body weight
cm: centimeter(s)
DBIL: direct bilirubin
dL: deciliter(s)
EDV: end‐diastolic volume
EDVn: normalized end‐diastolic volume
ESV: end‐systolic volume
ESVn: normalized end‐systolic volume
g: gram(s)
g/dL: gram(s) per deciliter
H&E: hematoxylin and eosin
HBT: heart‐base tumor
IVSd: interventricular septal diameter in diastole
IVSs: interventricular septal diameter in systole
kg: kilogram(s)
LA: left atrium/left atrial
LA/Ao: left atrium to aorta ratio
LAD: left atrial diameter
LADn: normalized left atrial diameter
LAu: left auricle
LVIDd: left ventricular internal diameter in diastole
LVIDdN: normalized left ventricular internal diameter in diastole
LVIDs: left ventricular internal diameter in systole
LVIDsN: normalized left ventricular internal diameter in systole
LVPWd: left ventricular posterior wall diameter in diastole
LVWd: left ventricular wall diameter in diastole
LVWs: left ventricular wall diameter in systole
mg/dL: milligram(s) per deciliter
MHz: megahertz
mL: milliliter(s)
mL/kg: milliliter(s) per kilogram
mm: millimeter(s)
PCV: packed cell volume
PE: pericardial effusion
RA: right atrium/right atrial
RV: right ventricle
TBIL: total bilirubin
TP: total protein
U/L: unit(s) per liter

## Author Contributions

S.P. performed echocardiography and pericardiocentesis and drafted the manuscript. A.M. and S.R. performed the necropsy and histopathological examination. M.R. and A.S. assisted in clinical management of the patient. This dog was a resident of Khodiyar Animal Welfare Trust, Jamnagar, Gujarat, India.

## Funding

No funding was received for this manuscript.

## Disclosure

The work was carried out as part of the authors′ routine clinical duties at Greens Zoological Rescue and Rehabilitation Centre and Khodiyar Animal Welfare Trust. All authors read and approved the final manuscript.

## Consent

Written consent for the diagnostic and therapeutic procedures described, and for the use of clinical data and images for scientific publication, was obtained from the authorized representative of Khodiyar Animal Welfare Trust.

## Conflicts of Interest

The authors declare no conflicts of interest.

## Data Availability

The data that support the findings of this study are available from the corresponding author upon reasonable request.
